# Advancements in Interventional Radiology for Managing Hepatic Encephalopathy: A Comprehensive Review

**DOI:** 10.7759/cureus.62723

**Published:** 2024-06-19

**Authors:** Shivani S Bothara, Pratapsingh Parihar, Ravishankar Patil

**Affiliations:** 1 Radiodiagnosis, Jawaharlal Nehru Medical College, Datta Meghe Institute of Higher Education and Research, Wardha, IND

**Keywords:** clinical outcomes, minimally invasive techniques, portal hypertension, tips procedure, interventional radiology, hepatic encephalopathy

## Abstract

Hepatic encephalopathy (HE) is a complex neuropsychiatric syndrome resulting from liver dysfunction, leading to cognitive, behavioral, and motor impairments. The management of HE has traditionally relied on pharmacological treatments, dietary modifications, and liver transplantation. However, recent advancements in interventional radiology (IR) have introduced minimally invasive procedures that offer promising alternatives. This comprehensive review explores the latest IR techniques, including transjugular intrahepatic portosystemic shunt (TIPS), balloon-occluded retrograde transvenous obliteration (BRTO), portal vein embolization (PVE), and Yttrium-90 (Y90) radioembolization. The efficacy, clinical outcomes, and potential complications of these techniques are examined through an analysis of current studies and trials. The review highlights the benefits of IR in reducing portal hypertension and improving hepatic blood flow, ultimately alleviating HE symptoms. Additionally, it underscores the importance of multidisciplinary collaboration, ongoing research, and the development of clear patient selection criteria to optimize the use of IR in HE management. By integrating these advancements into clinical practice, healthcare providers can enhance the quality of care and improve outcomes for patients with HE.

## Introduction and background

Hepatic encephalopathy (HE) is a neuropsychiatric syndrome characterized by cognitive, behavioral, and motor dysfunction in patients with liver disease. It arises from the accumulation of neurotoxic substances, particularly ammonia, due to impaired liver function. HE manifests along a spectrum of severity, ranging from subtle cognitive impairment to coma, posing significant challenges in diagnosis and management [1]. Interventional radiology (IR) plays a crucial role in the management of HE by offering minimally invasive procedures aimed at improving portal hypertension and reducing neurotoxicity. IR techniques, such as transjugular intrahepatic portosystemic shunt (TIPS) placement, balloon-occluded retrograde transvenous obliteration (BRTO), and portal vein embolization (PVE), provide effective strategies for addressing the underlying pathophysiology of HE, thus alleviating its symptoms and improving patient outcomes [2].

This comprehensive review aims to explore the advancements in IR techniques for managing HE. By examining the latest research findings, clinical outcomes, and future directions in the field, this review seeks to elucidate the role of IR in HE management and provide insights into potential therapeutic strategies. Additionally, the review will discuss the scope of IR interventions, their clinical efficacy, challenges, and opportunities for further research, thereby contributing to the broader understanding of HE management and enhancing clinical practice.

## Review

Traditional treatment approaches for HE

Pharmacological Interventions

**Figure 1:**
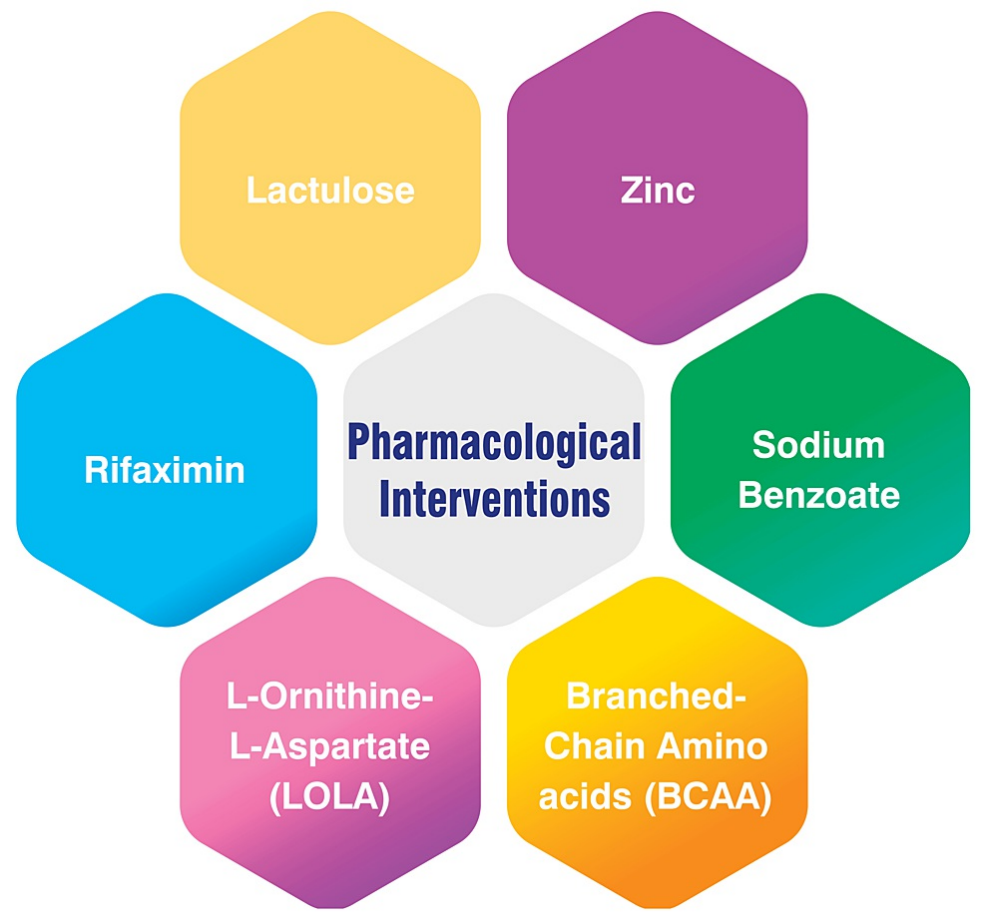
Pharmacological interventions Image Credit: Dr. Shivani Bothara

Several pharmacological interventions are commonly used to treat HE. Lactulose, an osmotic laxative, is recognized as a first-line treatment for overt HE due to its ability to reduce ammonia absorption and facilitate its excretion through the colon [3,4]. Rifaximin, a non-absorbable antibiotic, is often combined with lactulose for recurrent HE as it diminishes ammonia production by gut bacteria [3-5]. Another option is L-ornithine-L-aspartate (LOLA), a stable salt of ornithine and aspartic acid, which promotes urea and glutamine synthesis, potentially lowering blood ammonia levels. LOLA can be administered orally or intravenously [5]. Branched-chain amino acids (BCAA) may be beneficial in treating overt HE compared to placebo or no intervention, though not as effective as lactulose or neomycin [5,6]. Sodium benzoate, a preservative, conjugates with glycine to form hippuric acid, aiding in kidney excretion and reducing blood ammonia levels [6]. Additionally, zinc supplementation, often deficient in cirrhosis, can enhance urea cycle enzymes and mitigate oxidative stress, potentially improving HE symptoms [6]. These pharmacological interventions target different aspects of ammonia metabolism to reduce its production, increase elimination, and address underlying liver dysfunction. The selection of therapy depends on various factors, such as the severity of HE, patient characteristics, and response to treatment. More advanced interventions like TIPS reduction or shunt occlusion may be necessary in refractory cases to effectively manage the condition [5,7]. Pharmacological interventions are shown in Figure 1.

Dietary Management

**Figure 2:**
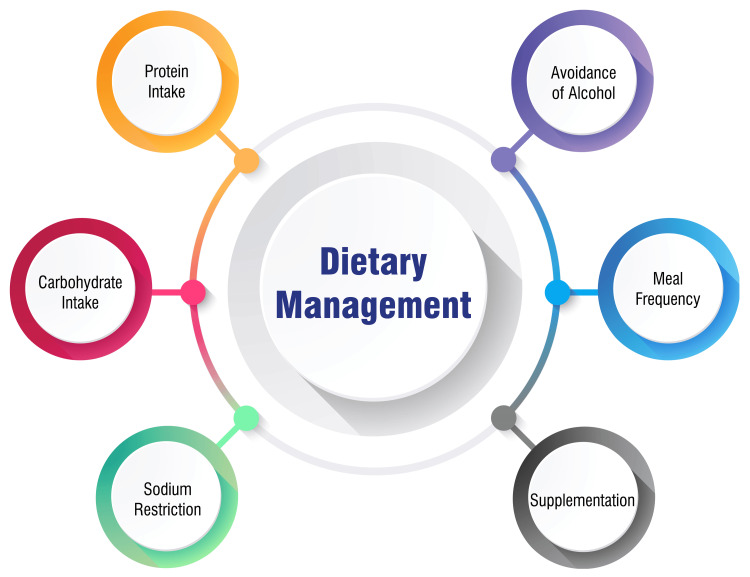
Dietary management Image Credit: Dr. Shivani Bothara

Dietary management plays a significant role in treating HE, with current guidelines emphasizing specific nutritional strategies to optimize patient outcomes. Contrary to practices advocating protein restriction, current guidelines recommend adequate protein intake ranging from 1.2 to 1.5 grams per kilogram of body weight per day. This protein should be sourced from high-quality, easily digestible sources such as dairy, eggs, and fish. Severe protein restriction can lead to muscle wasting and exacerbate HE symptoms [8,9]. Increasing complex carbohydrate intake from sources like whole grains, fruits, and vegetables is recommended to provide sufficient calories and decrease reliance on protein for energy. This dietary adjustment helps maintain a balanced nutrient intake while supporting metabolic processes [10]. Patients with fluid retention and ascites are advised to limit sodium intake to prevent further fluid accumulation. Sodium restriction is crucial in managing fluid balance and minimizing the risk of complications associated with fluid overload [10]. Supplementation with specific nutrients like BCAA, LOLA, and probiotics may offer benefits in managing HE by reducing ammonia levels and improving overall nutritional status. These supplements target the key metabolic pathways of ammonia detoxification and the balance of gut microbiota [5,11]. Opting for smaller, more frequent meals rather than large, infrequent ones helps prevent prolonged fasting, which can exacerbate HE symptoms. This meal frequency strategy supports steady energy provision and minimizes fluctuations in metabolic processes [10]. Alcohol consumption should be completely avoided in patients with HE, as it can further compromise liver function and worsen HE symptoms. Eliminating alcohol intake is essential for preserving liver health and optimizing treatment outcomes [10]. The primary goal of dietary management in HE is to provide adequate nutrition, reduce ammonia production, and improve the patient's nutritional status. Implementing these dietary strategies alongside medical interventions can contribute to better outcomes and enhance the management of HE [5,11]. Dietary management is shown in Figure 2.

Liver Transplantation

Liver transplantation is a surgical procedure utilized to replace a patient's diseased or failing liver with a healthy liver obtained from either a deceased donor or a living donor. It stands as a pivotal life-saving intervention for individuals grappling with end-stage liver disease or acute liver failure [12]. Among adults, common indications for liver transplantation encompass alcoholic liver disease, liver cancer, non-alcoholic steatohepatitis (fatty liver disease), and cirrhosis resulting from chronic hepatitis C. In pediatric cases, biliary atresia predominates as the primary reason for liver transplantation [13]. The intricate process of liver transplantation involves the meticulous removal of the patient's diseased liver, followed by the transplantation of the donor liver. The donor's liver is carefully implanted and meticulously connected to the patient's blood vessels and bile ducts. This complex surgical procedure typically demands approximately 5-6 hours to complete [14].

While liver transplantation offers profound benefits, it also carries inherent risks. Potential complications encompass primary non-function of the graft, hepatic artery thrombosis, portal vein thrombosis, biliary complications, and infection susceptibility. Thus, careful patient selection, donor-recipient matching, and meticulous surgical techniques are imperative in achieving favorable outcomes [15]. Liver transplantation boasts remarkable long-term survival rates. For deceased donor transplants, the one-year survival rate approximates 90%, with the five-year survival rate hovering around 77% [16]. Additionally, living donor liver transplantation has emerged as a critical alternative, particularly in regions constrained by limited deceased donor availability [16]. The successful management of liver transplant patients necessitates the collaborative efforts of an interprofessional healthcare team comprising surgeons, hepatologists, nurses, and various other healthcare providers. Their collective expertise is indispensable, guiding patients through every stage of the transplant journey- from pre-transplant evaluation to post-operative care and long-term follow-up [16].

Role of IR in HE management

Overview of IR Techniques

IR encompasses a spectrum of minimally invasive procedures guided by sophisticated medical imaging modalities such as X-rays, CTs, MRIs, and ultrasounds. These procedures utilize specialized tools like needles, guidewires, sheaths, and catheters to access internal structures through small incisions or body orifices, facilitating diagnostic and therapeutic interventions. Diagnostic IR techniques are pivotal in aiding diagnosis and guiding subsequent treatments, encompassing procedures like angiography for visualizing blood vessels, cholangiography for imaging bile ducts, and biopsies for obtaining tissue samples for analysis [17]. On the therapeutic front, interventional radiologists execute diverse procedures to address various conditions directly. Examples include angioplasty and stenting to alleviate blocked blood vessels, kyphoplasty for treating spinal fractures, embolization to obstruct blood flow to tumors or abnormal vessels, ablation to eradicate tumors or abnormal tissue, and dialysis access management for the maintenance and repair of fistulas and grafts. These interventions are performed precisely under real-time imaging guidance, ensuring accuracy while minimizing risks typically associated with conventional open surgeries [18]. The hallmark advantages of IR stem from its minimally invasive nature, which translates into reduced pain, shorter recovery times, and decreased risks of complications compared to traditional surgical approaches. Leveraging cutting-edge imaging technologies and innovative techniques, interventional radiologists can effectively address a broad spectrum of conditions, spanning vascular disorders, oncological diseases, and numerous other medical issues. Continual advancements in IR provide patients with increasingly less invasive yet highly effective alternatives to conventional surgical procedures, marking a significant stride in modern medicine [19].

Current Practices and Challenges

IR has evolved significantly, offering minimally invasive procedures for diagnosing and treating various medical conditions. However, despite its potential, IR encounters several challenges that impede its growth and the effective delivery of services [20]. One of the primary hurdles in IR is the training of interventional radiologists. The absence of a distinct identity for IR makes it challenging to quantify the number of interventional radiologists accurately. This lack of clarity in defining the specialty leads to inadequate workforce planning, affecting the availability of expertise and hindering specialty development [21]. Another significant challenge lies in the non-clinical pattern of practice observed in IR. This emphasis on technical aspects can obscure patient pathways and reduce referrals for IR procedures. To mitigate this issue, interventional radiologists must assume full clinical responsibility for patient care. However, this is often impeded by the influence of other clinicians on patient pathways [22]. In numerous countries, including the UK, the IR workforce is insufficient to meet basic clinical demands. Poor recruitment from within radiology and the absence of a distinct identity for IR exacerbate this shortage. The current rate of trainee growth in IR is inadequate to fulfill the requirement for a sufficient number of interventional radiologists to fill consultant posts in the foreseeable future [21,22]. Interventional radiologists employ minimally invasive techniques to diagnose and treat various conditions, including abnormal connections between arteries and veins. Common procedures encompass angioplasty and stenting, kyphoplasty, embolization, and ablation, frequently used in treating vascular disorders and oncological diseases [23].

Potential Advantages Over Traditional Treatments

IR procedures offer a multitude of benefits compared to traditional surgery. First, these procedures are typically performed under local anesthesia, significantly reducing the risk of pain and discomfort associated with conventional surgery [24,25]. Second, minimally invasive techniques in IR lead to faster recovery times, enabling patients to resume their daily activities within days rather than weeks [25,26]. Additionally, the minimally invasive nature of IR procedures results in lower risks of complications and infection, enhancing patient safety [25,27]. Another advantage is the potential for outpatient treatment, as many IR procedures can be performed without hospital stays. This minimizes inconvenience for patients and reduces the overall cost of treatment [24,25]. Moreover, IR has demonstrated superior patient safety and satisfaction outcomes. With less invasive procedures and local anesthesia, the risks associated with general anesthesia are mitigated, contributing to better overall outcomes [25,26]. Furthermore, IR procedures are often quicker than traditional surgery, allowing for more efficient use of medical resources and reducing treatment costs [25]. Finally, IR empowers patients by offering them more options and choices regarding their treatment. This patient-centered approach aligns with their health goals and lifestyles, fostering a sense of control and involvement in their care [25].

Advancements in IR techniques for HE

*TIPS* *Procedure*

The TIPS procedure, conducted by an interventional radiologist, is a minimally invasive technique designed to establish a connection between the portal vein and the hepatic vein within the liver [28,29]. This connection serves to circumvent abnormal liver pathways and alleviate elevated blood pressure in the portal vein. TIPS is primarily employed in the treatment of complications arising from portal hypertension, including variceal bleeding, portal hypertensive gastropathy, and severe ascites [29,30]. Additionally, it may be utilized in select cases of Budd-Chiari syndrome. The procedure entails the insertion of a catheter through the jugular vein, meticulously guiding it to the liver, and subsequently utilizing a needle to create a passage between the portal and hepatic veins. Subsequently, a stent is deployed to maintain the patency of this passage [28,29]. Potential complications associated with TIPS include HE and heart failure. Therefore, diligent patient selection and management are imperative to mitigate these risks [29,30]. TIPS has emerged as a less invasive alternative to traditional surgical shunts for addressing complications related to portal hypertension. When executed within appropriate clinical contexts, the benefits of TIPS often outweigh the potential risks associated with the procedure [30].

BRTO

BRTO represents an advanced IR approach to addressing gastric varices and portosystemic shunts. This technique entails the occlusion of outflow veins, such as the gastrorenal shunt, employing an occlusion balloon, followed by the direct injection of a sclerosing agent into the varix endovascularly. BRTO aims to modulate blood flow within the varix, facilitating the stagnation of sclerosant material within the gastric varix without reflux into other vasculature. Consequently, this process induces thrombosis and scarring within the variceal system [31,32]. BRTO demonstrates particular efficacy in managing gastric variceal bleeding, a critical complication of portal hypertension, and can serve as an alternative or adjunct to transjugular intrahepatic shunts (TIPS) in specific scenarios. Notably, the technique exhibits high technical success rates and proves beneficial in emergency and prophylactic treatment of gastric varices. Moreover, various modifications of BRTO, including combined procedures with partial splenic embolization, show promise in mitigating the exacerbation of esophageal varices and enhancing patient outcomes [31,33]. In essence, BRTO is a valuable endovascular technique that plays a pivotal role in managing gastric varices and portosystemic shunts. By offering a minimally invasive and effective treatment option, BRTO significantly improves the outcomes of patients afflicted with these conditions.

PVE

PVE is a preparatory procedure conducted in IR to stimulate hypertrophy of the liver remnant ahead of planned hepatic resection. This technique entails injecting embolic material into the portal vein, thereby obstructing blood flow to specific liver areas earmarked for preservation. Consequently, blood is redirected to healthy liver segments, fostering hyperplasia. PVE is recommended when the future liver remnant (FLR) to total estimated liver volume (TELV) ratio is deemed insufficient for safe resection, with recommended FLR/TELV ratios typically set at a minimum of 25% in normal livers and 40% in cases involving chronic liver disease such as cirrhosis [34]. The procedure is generally well-tolerated, characterized by low mortality rates and minimal complications, including portal vein thrombosis, liver infarction, and infection. PVE offers notable benefits, including reduced post-resection morbidity, facilitation of resections in initially unresectable tumors, and decreased post-resection mortality rates by augmenting functional liver volume. Additionally, PVE aids in predicting compensatory regeneration following liver resection, thus contributing to improved surgical outcomes by averting liver insufficiency and promoting liver regeneration [35,36]. However, PVE does entail certain contraindications, including portal hypertension, complete lobar portal vein occlusion, extrahepatic metastatic disease, and an inadequate predicted FLR post-PVE. Risks associated with PVE encompass portal vein thrombosis, liver infarction, necrosis, and those inherent to percutaneous transhepatic procedures. Despite these risks, PVE remains a valuable technique for managing patients with insufficient FLR/TELV ratios, offering them a pathway to safer hepatic resections and enhanced postoperative outcomes [35,36].

Yttrium-90 Radioembolization Therapy

Yttrium-90 (Y90) radioembolization is a minimally invasive and targeted approach to treating liver tumors, combining embolization and radiation therapy. This procedure entails injecting minuscule radioactive beads, known as microspheres, into the blood vessels supplying the tumor. It is predominantly utilized in treating liver cancers, encompassing hepatocellular carcinoma (HCC) and metastatic tumors originating from other body regions [37-40]. Y90 radioembolization serves as an alternative treatment avenue for patients grappling with liver cancer that is not amenable to surgical intervention or liver transplantation [37]. The radioactive Y90 isotope emits beta radiation, which traverses a short distance and primarily targets cancer cells while sparing healthy liver tissue [38,39]. Concurrently, the microspheres obstruct the blood supply to the tumor, further contributing to its demise [37-40]. Research findings underscore the efficacy of Y90 radioembolization in prolonging the lives of patients with inoperable liver tumors and enhancing their overall quality of life [37-40]. Despite these advancements, the prognosis for liver cancer remains challenging, with five-year survival rates ranging from 3% for distant stage to 36% for localized disease [37]. Factors associated with a less favorable outcome for Y90 radioembolization include advanced age, later cancer stage, and history of portal vein thrombosis [37]. Ongoing clinical trials aim to elucidate the comparative effectiveness of Y90 radioembolization against alternative treatment modalities, such as sorafenib and transarterial chemoembolization (TACE) [37].

Clinical efficacy and outcomes of IR in HE

Review of Clinical Studies and Trials

IR procedures, such as TIPS and spontaneous portosystemic shunts (SPSS), play a pivotal role in the development of HE, particularly among patients with chronic liver disease [41]. Techniques like shunt occlusion and TIPS reduction have shown efficacy and safety in alleviating neurological symptoms in individuals with refractory HE, focusing on managing portal-systemic shunts integral to its pathophysiology [41,42]. A review of ongoing clinical trials in HE identified 17 active studies as of August 2022, with over 75% of these trials in phase II (41.2%) or phase III (34.7%) stages [1]. These trials encompass therapeutic agents, from established drugs like lactulose and rifaximin to innovative approaches such as fecal microbiota transplantation and immunosuppressive agents [41]. Additionally, some trials explore therapies borrowed from other medical conditions, including antimicrobial agents and microbiome restoration therapies [41]. The West Haven Criteria, a commonly utilized classification system, categorizes HE based on levels of consciousness and cognitive/behavioral assessments, ranging from mild to severe manifestations [43,44]. Diagnosis entails a battery of tests encompassing liver function evaluation, brain imaging studies, and assessment of ammonia levels [43,44]. Symptoms of HE span a spectrum from mild forgetfulness and irritability to severe confusion, seizures, and coma if left unaddressed [43,44]. The search results underscore the burgeoning interest and advancements in IR techniques for managing HE, particularly by targeting portal-systemic shunts. Furthermore, the diverse pipeline of potential therapies highlighted by ongoing clinical trials, comprising both repurposed and novel agents, offers promise for enhanced treatment options for this intricate neuropsychiatric complication of liver disease.

Complications and Adverse Events

Complications within IR represent a subset of adverse events characterized by any deviation from the normal post-therapeutic course, whether symptomatic or asymptomatic [45]. While the majority of IR procedures are successful, complications can result in significant morbidity, necessitating more invasive corrective measures and exposing patients to increased cumulative risk [45]. Contributing factors to complications in IR encompass the complexity of the procedure, inherent patient factors, and the techniques utilized. For instance, complications linked to arterial access in coronary interventions can arise in up to 9% of cases, encompassing issues such as hematoma, pseudoaneurysm, hemorrhage, and arteriovenous fistula formation [45]. Many complications are potentially preventable through optimizing and standardizing techniques, alongside a deeper understanding of how patient risk factors and procedural factors influence outcomes [45,46]. Efforts are underway to cultivate a robust safety culture within IR through quality improvement initiatives, adherence to best-practice guidelines, and the implementation of revised classification and reporting schemas for adverse events [45,47]. However, the true prevalence and underlying causes of complications in IR remain inadequately understood, primarily due to a relative scarcity of healthcare registries and investigative literature compared to other medical specialties [46,47]. Complications should not be viewed in isolation but should be considered within the broader context of clinical governance and the intricate healthcare system in which they arise [47]. Ultimately, complications constitute an inevitable aspect of clinical practice, necessitating a systematic, transparent, and conscientious approach to their management to attain optimal patient outcomes in IR [47].

Future directions and emerging technologies

Innovations in IR

The integration of advanced imaging modalities, such as digital angiography, portable ultrasound units, and 4D-CT, has significantly enhanced the precision and safety of IR procedures. These advanced imaging techniques improve diagnostic accuracy and enable real-time visualization during interventions, thereby enhancing procedural outcomes [48]. Furthermore, substantial advancements in intravascular stents, including balloon-mounted, self-expanding, covered stents, and flow diversion stents, have revolutionized the treatment of vascular conditions. Developing intravascular filters and embolic agents has further improved patient outcomes and safety in IR procedures [48]. The transition toward personalized medicine and using biomarkers in IR have facilitated tailored treatment strategies based on individual patient characteristics. This personalized approach enhances the efficacy of interventions and optimizes patient outcomes [48]. Moreover, the evolution of minimally invasive techniques, such as TIPS and SPSS occlusion, has significantly reduced complications and improved patient recovery. These scarless treatment options offer effective solutions for various conditions, further advancing the field of IR [49]. Integrating artificial intelligence and robotics into IR procedures holds great promise for enhancing precision, reducing radiation exposure, and improving procedural outcomes. These innovative technologies are expected to play a pivotal role in shaping the future of IR [50]. Last, the emphasis on collaboration between interventional radiologists, other medical specialties, and industry partners has fostered innovation and creativity in developing new treatment approaches. This multidisciplinary approach has facilitated the dissemination of novel procedures across different medical fields, ultimately enhancing patient care and outcomes [50].

Potential Areas for Further Research

Developing personalized treatment strategies tailored to individual patient characteristics and biomarkers could significantly enhance the efficacy of IR techniques. This approach may involve identifying specific patient subgroups most likely to benefit from certain interventions, thereby optimizing treatment outcomes [51]. Integrating advanced imaging modalities, such as functional MRI, diffusion tensor imaging, and magnetic resonance spectroscopy, into the diagnostic and monitoring processes of HE could improve the accuracy and early detection of encephalopathy. These advanced techniques have the potential to identify early signs of encephalopathy and track the effectiveness of interventions more effectively [52]. Further refinement of minimally invasive procedures like TIPS and SPSS occlusion aims to reduce complications and enhance patient outcomes. Tailoring these interventions to individual patient needs and exploring local anesthesia could minimize risks and improve procedural success rates [53]. Investigating the benefits of combining IR techniques with other treatment modalities, such as pharmacological interventions and nutritional management, may enhance the effectiveness of managing HE. This approach could offer synergistic effects and improve patient outcomes [51,53]. Exploring the integration of robotic technology into IR procedures has the potential to enhance precision and reduce the risk of complications, particularly in complex procedures like shunt occlusion and TIPS reduction. Robotic assistance could improve procedural outcomes and patient safety [51,53]. Applying artificial intelligence and machine learning algorithms to analyze large datasets could optimize treatment strategies for individual patients, improving the accuracy and efficiency of IR procedures. These technologies promise to enhance treatment outcomes and patient care [54]. Developing new materials and devices for IR procedures, such as improved stents and coils, could enhance the effectiveness and safety of treatments for HE. Advancements in device technology may lead to better outcomes and reduced procedural risks [54]. Utilizing patient-specific modeling and simulation techniques could enhance the planning and execution of IR procedures, allowing for more precise and effective interventions. Patient-specific simulations enable clinicians to anticipate challenges and optimize treatment strategies beforehand [51]. Integrating real-time monitoring and feedback systems into IR procedures could provide immediate feedback on the effectiveness of interventions and enable adjustments during the procedure. Real-time feedback enhances procedural safety and efficacy, improving patient outcomes [51]. Fostering multidisciplinary collaboration between interventional radiologists, hepatologists, and other specialists is essential to identify the most effective treatment strategies and ensure comprehensive care for patients with HE. Collaborative efforts facilitate the exchange of expertise and resources, improving patient outcomes [54].

Integration With Multidisciplinary Approaches

IR fosters collaboration among healthcare professionals from diverse disciplines, including hepatologists, oncologists, and surgeons, to deliver comprehensive and personalized care for patients [55]. This collaborative approach ensures that each patient's unique needs are thoroughly addressed, leading to more effective treatment outcomes. Working closely with primary care physicians and other specialists, interventional radiologists facilitate specialized care through referrals, ensuring patients receive integrated and seamless healthcare services without the need to navigate between multiple specialists [56]. This collaborative model enhances the continuity of care and improves patient satisfaction. In the management of complex conditions like complicated pancreatitis, IR plays a longitudinally integrated role, contributing to clinical decision-making at various stages of treatment. This comprehensive understanding of the patient's journey enables interventional radiologists to provide tailored interventions and optimize patient outcomes [56]. Active participation in multidisciplinary tumor boards and conferences allows interventional radiologists to collaborate with other specialists in developing consensus-based treatment plans for cancer patients. This collective approach ensures the selection of the most appropriate and effective interventions tailored to each patient's unique circumstances [55]. In palliative care settings for cancer patients, IR plays a crucial role alongside medical therapy and psychological support. Integrating IR into multidisciplinary palliative care teams enables effective symptom management and enhances the overall quality of life for patients [55]. The evolving landscape of diagnostic imaging and the growth of IR have led to increased radiologist involvement in patient management. Radiologists are now expected to actively participate in multidisciplinary teams, contributing their expertise to clinical decision-making processes and ensuring optimal patient care [55].

## Conclusions

In conclusion, the advancements in IR have markedly enhanced the management of HE, offering new hope for patients suffering from this debilitating condition. Techniques such as TIPS, BRTO, PVE, and Y90 radioembolization have demonstrated significant efficacy in reducing portal hypertension, improving hepatic blood flow, and alleviating HE symptoms. These minimally invasive approaches not only expand treatment options but also promote quicker recovery and better overall outcomes. For the future, ongoing research, comprehensive clinical trials, and the development of clear patient selection criteria are essential to further optimize these interventions. Additionally, fostering multidisciplinary collaboration and enhancing education and training for healthcare providers will ensure the effective integration of IR techniques into standard HE management protocols. Through these efforts, the clinical practice of managing HE can be significantly advanced, leading to improved patient care and quality of life.
